# Short-Term Residual Myopic Bias Measured by Open-Field Autorefraction After 10-Second Myopic Overcorrection in Young Myopic Adults

**DOI:** 10.3390/vision10030047

**Published:** 2026-07-24

**Authors:** Yo Iwata

**Affiliations:** Department of Rehabilitation, Orthoptics and Visual Science Course, School of Allied Health Sciences, Kitasato University, 1-15-1 Kitasato, Sagamihara 252-0373, Japan; iwatayo@kitasato-u.ac.jp; Tel.: +81-42-778-9667

**Keywords:** accommodation, autorefractor, myopia, overcorrection, subjective refraction

## Abstract

The magnitude and short-term recovery of residual objective myopic bias after a brief, clinically plausible over-minus exposure during distance refraction remain insufficiently quantified. This study quantified the short-term residual objective myopic shift following 10 s of transient myopic overcorrection of 0.50 D or 1.00 D in young adults with myopia. Twenty healthy young adults with myopia and low astigmatism underwent open-field automated objective over-refraction (OR) while viewing a high-contrast 5 m target. After trial lenses induced a moderate myopic state, each participant completed three randomized 10 s lens conditions: 0.50 D undercorrection, 0.50 D overcorrection, and 1.00 D overcorrection. The primary endpoint was OR at 5 s after restoration of the objective neutral correction. Both overcorrection conditions produced significantly more negative OR than undercorrection at 5 s; exploratory analyses showed the same pattern at 10 s. The shift was evident at 5 and 10 s after restoration of the objective neutral correction and, by 30 s, was no longer statistically significant and appeared clinically negligible. This bias should be considered when OR measurements obtained shortly after over-minus lens exposure are used to inform subsequent subjective refinement.

## 1. Introduction

The global prevalence of refractive error, especially myopia, continues to increase, and approximately half of the global population is projected to have myopia by 2050 [1,2]. Appropriate refractive correction is therefore essential, and the demand for spectacles and contact lenses is expected to increase accordingly.

Accurate subjective refraction is required to determine the corrective power. However, myopic overcorrection and other refractive mismatches can cause blurred vision, asthenopia [3], headache [4], accommodative spasm [5], unstable binocular vision [6], and reduced contrast sensitivity [7]. These symptoms can reduce spectacle or contact lens acceptance and subjective satisfaction [8,9] and may be associated with other adverse effects.

During subjective refraction in patients with myopia, refinement should proceed stepwise from lower minus powers toward the least minus lens that provides the best visual acuity [10,11,12,13]. This approach reflects accommodative dynamics: accommodation generally relaxes rapidly, but a smaller, slower component can persist for several to tens of seconds [14,15]. Because young adults can maintain good visual acuity even when slightly overcorrected, inadvertent myopic overcorrection during refraction is possible.

Objective refraction from an autorefractor is commonly used as a starting point in clinical practice; nevertheless, subjective refinement in myopic eyes is typically initiated with minus powers weaker than the objective refraction value [16,17]. Accommodative adaptation, tonic accommodation, and near-work-induced transient myopia (NITM) are established phenomena, and the present study was not designed to propose a new physiological mechanism. Instead, this study addressed a narrower clinical measurement question: whether a brief, clinically plausible 10 s over-minus exposure during distance refraction leaves a measurable residual objective myopic bias after restoration of the objective neutral correction. Specifically, this study aimed to quantify the magnitude and short-term time course of objective over-refraction (OR) after 0.50 D and 1.00 D transient myopic overcorrection in young adults with myopia.

This time-course information may help guide the timing of OR measurements after transient over-minus exposure during clinical refraction.

## 2. Materials and Methods

### 2.1. Study Design and Participants

This exploratory, randomized, within-subject crossover pilot study was conducted in young adults with myopia. Each participant completed all three nominal lens conditions in randomized order during a single experimental session.

Twenty healthy young adults with myopia were enrolled; only the right eye of each participant was analyzed. Participants with astigmatism of 1.00 D or greater or organic ocular disease were excluded. Participant characteristics are reported in Section 3. Axial length was not measured because the present pilot study was designed to quantify the short-term within-subject objective refractive bias after brief myopic overcorrection, rather than to evaluate structural determinants of myopia or accommodation. Baseline accommodative and binocular vision metrics, including amplitude of accommodation, phoria status, and convergence measures, were not measured.

The predefined sample size was 20 participants. No formal a priori sample size calculation was performed. The sample size was selected to estimate the within-subject magnitude and short-term time course of residual objective myopic bias after brief myopic overcorrection and to inform future confirmatory studies.

The study complied with the Declaration of Helsinki and was approved by the Japan Orthoptic Vision Society Human Sciences Ethics Committee (approval No. JOVS-25004; approved on 10 February 2025). Written informed consent was obtained from all participants. Participants were recruited between 10 February 2025, and 21 December 2025.

### 2.2. Measurements

Objective refraction was recorded while participants fixated on a target at 5 m. The fixation target was a Landolt C (gap oriented upward) with a size of 0.3 logMAR, and it was presented on a fourth-generation iPad Pro (Apple Inc., Cupertino, CA, USA; 2732 × 2048 pixels, 264 ppi). Refraction was measured using an open-field autorefractor (WAM-5500; Grand Seiko Co., Ltd., Fukuyama, Japan) at a sampling frequency of 5 Hz. In this protocol, OR was recorded after restoration of the objective neutral correction at 5, 10, and 30 s. The WAM-5500 has been reported to show good clinical performance and repeatability [18,19]. The fellow eye (left) was covered with an opaque occluder mounted on the trial frame throughout each experimental condition, including the 10 s lens-exposure period and all subsequent right-eye OR measurements. Thus, both the lens-exposure and measurement periods were performed under monocular viewing conditions. The measurement increment was 0.01 D, and the trial lenses were available in 0.05 D increments. The room illuminance was maintained at 600–800 lx.

In this study, OR referred to the measured spherical-equivalent refractive error through trial lenses while participants viewed the 5 m target. OR was treated as the measured value, whereas the 10 s lens conditions were treated as imposed nominal lens states. The objective neutral correction was defined as the trial-lens condition in which the measured OR was 0.00 D during distance fixation. A negative OR indicated residual myopia relative to the objective neutral correction, whereas a positive OR indicated an over-minus state relative to the objective neutral correction. After the objective neutral correction was restored, negative OR values at 5, 10, or 30 s were interpreted as residual objective myopic bias. OR was not recorded during the 10 s nominal lens-exposure period.

Each participant was first placed in an initial residual myopic state by adding trial lenses until the measured OR was −1.00 D. From this initial −1.00 D OR state, additional trial lenses were inserted to create one of the following three 10 s nominal lens conditions:

1. 0.50 D undercorrection. From the initial −1.00 D OR state, −0.50 D of additional minus power was inserted, corresponding to an intended nominal OR of −0.50 D based on the lens change. Participants fixated on the 5 m target under this condition for 10 s. The objective neutral correction was then restored by adding an additional −0.50 D, and OR was measured at 5, 10, and 30 s during the subsequent 30 s of distance fixation.
2. 0.50 D overcorrection. From the initial −1.00 D OR state, −1.50 D of additional minus power was inserted, corresponding to an intended nominal OR of +0.50 D based on the lens change. Participants fixated on the 5 m target under this condition for 10 s. The objective neutral correction was then restored by adding +0.50 D, and OR was measured at 5, 10, and 30 s.
3. 1.00 D overcorrection. From the initial −1.00 D OR state, −2.00 D of additional minus power was inserted, corresponding to an intended nominal OR of +1.00 D based on the lens change. Participants fixated on the 5 m target under this condition for 10 s. The objective neutral correction was then restored by adding +1.00 D, and OR was measured at 5, 10, and 30 s.

**Figure 1:**
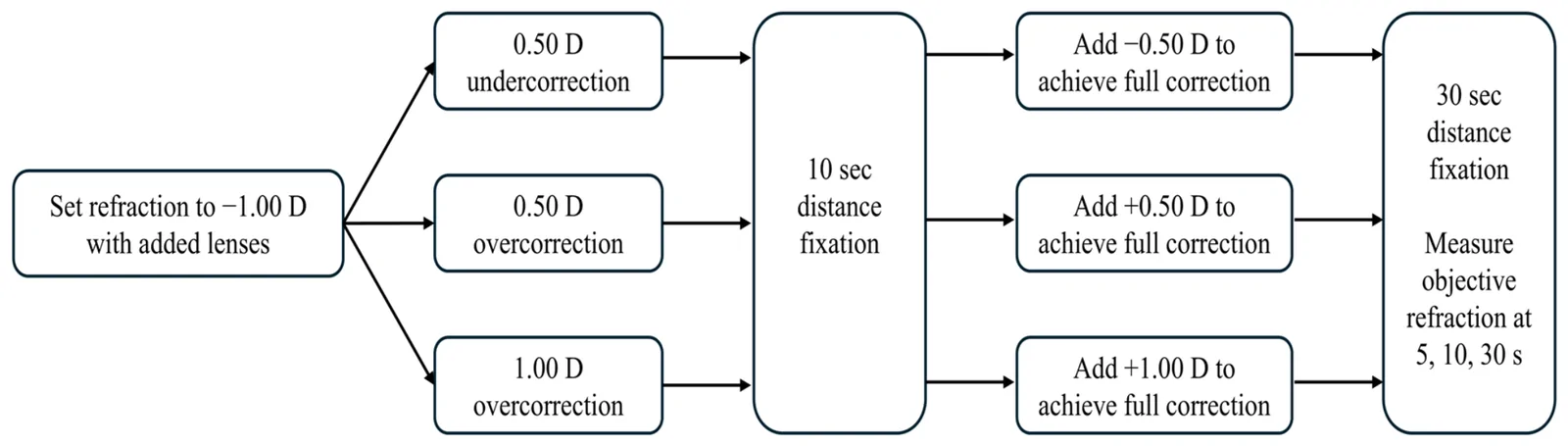
Schematic diagram of the experimental procedure used in this study.

The mean of three consecutive measurements was used for each time point. The order of the conditions was randomized for each participant using the RAND function in Microsoft Excel for Microsoft 365 (Microsoft Corporation, Redmond, WA, USA). All three conditions were completed in a single session. To reduce carryover between conditions, participants rested for 5 min between conditions after trial-lens removal while viewing a distant target. Before the next condition began, OR was confirmed to have returned to approximately 0.00 D. The protocol is provided in Figure 1.

### 2.3. Statistical Analyses

For statistical analysis, data were treated as paired within-subject repeated measurements. The primary endpoint was OR at 5 s after restoration of the objective neutral correction. The primary analysis compared OR among the three nominal lens conditions at this 5 s time point: 0.50 D undercorrection, 0.50 D overcorrection, and 1.00 D overcorrection. Three prespecified paired comparisons were performed for the primary endpoint: 0.50 D overcorrection versus 0.50 D undercorrection, 1.00 D overcorrection versus 0.50 D undercorrection, and 1.00 D overcorrection versus 0.50 D overcorrection. Because the sample size was small and normality could not be assumed, Wilcoxon signed-rank tests were used. The Holm procedure was applied to adjust for these three primary paired comparisons.

OR at 10 and 30 s after restoration of the objective neutral correction and within-condition temporal changes from 5 to 10 s and from 5 to 30 s were treated as secondary, exploratory outcomes. For the exploratory inter-condition comparisons at 10 and 30 s, the same three paired contrasts were examined at each time point, and Holm adjustment was applied separately within each time point. These analyses were used to describe the short-term time course of residual objective myopic bias and were not treated as independent confirmatory primary tests.

For both primary and exploratory paired comparisons, paired mean and median differences in OR between conditions were reported together with 95% bootstrap confidence intervals for the paired mean difference. For each comparison, the paired difference was calculated as the OR in the comparison condition minus the OR in the reference condition. Thus, negative paired differences indicated a more negative residual objective myopic bias in the comparison condition. Matched-pairs rank-biserial correlation was reported as an effect-size estimate. Bootstrap confidence intervals were estimated using 10,000 resamples of the paired observations. Exploratory within-condition analyses assessed changes over time using paired Wilcoxon signed-rank tests for 5 versus 10 s and 5 versus 30 s. The two tests within each condition were treated as one family and adjusted with the Holm method. All tests were two-sided, and statistical significance was set at *p* < 0.05. Descriptive statistics are reported as mean ± SD. Paired effects are reported as mean differences, median differences, 95% bootstrap confidence intervals, and matched-pairs rank-biserial correlations. To evaluate the adequacy of the sample size for the null inter-condition findings at 30 s, a post hoc sensitivity/power analysis was performed for the three paired 30 s comparisons. A clinically meaningful paired mean difference was defined as 0.25 D, corresponding to one conventional clinical lens step. Power was calculated from the observed standard deviation of the paired differences using a two-sided paired-samples framework. Power was evaluated at α = 0.05 and, as a conservative sensitivity check for the three 30 s comparisons, at α = 0.0167. Exploratory sequence-effect analyses were also performed to assess whether condition order influenced OR. For each time point, period/order effects were evaluated using a condition-adjusted repeated-measures model with OR as the outcome, current lens condition and measurement period as predictors, and participant as the clustering unit. Previous-condition effects were assessed among the second and third periods by adding the immediately preceding lens condition to a model that included the current lens condition and measurement period. The analyses were performed using BellCurve for Excel version 4.06 (Social Survey Research Information, Tokyo, Japan).

## 3. Results

Twenty participants (one right eye per participant) were included in the analysis. The mean age was 20.2 ± 0.8 years, and the cohort included four men and 16 women. Baseline spherical equivalent refraction was −3.25 ± 1.20 D, with a range of −1.25 to −5.75 D. Baseline astigmatism magnitude was 0.42 ± 0.24 D, with a range of 0.00 to 0.75 D.

The magnitude of the primary and exploratory inter-condition differences is summarized in Table 1. The primary endpoint was OR at 5 s after restoration of the objective neutral correction. Inter-condition comparisons at 10 and 30 s were treated as secondary exploratory analyses to describe the short-term recovery pattern. Negative paired differences indicate that OR values were more negative in the comparison condition than in the reference condition.

The 5 s comparisons were prespecified as the primary comparisons. The 10 s and 30 s comparisons were secondary exploratory analyses. Holm-adjusted *p* values were calculated within each time point for the three paired comparisons.

**Figure 2:**
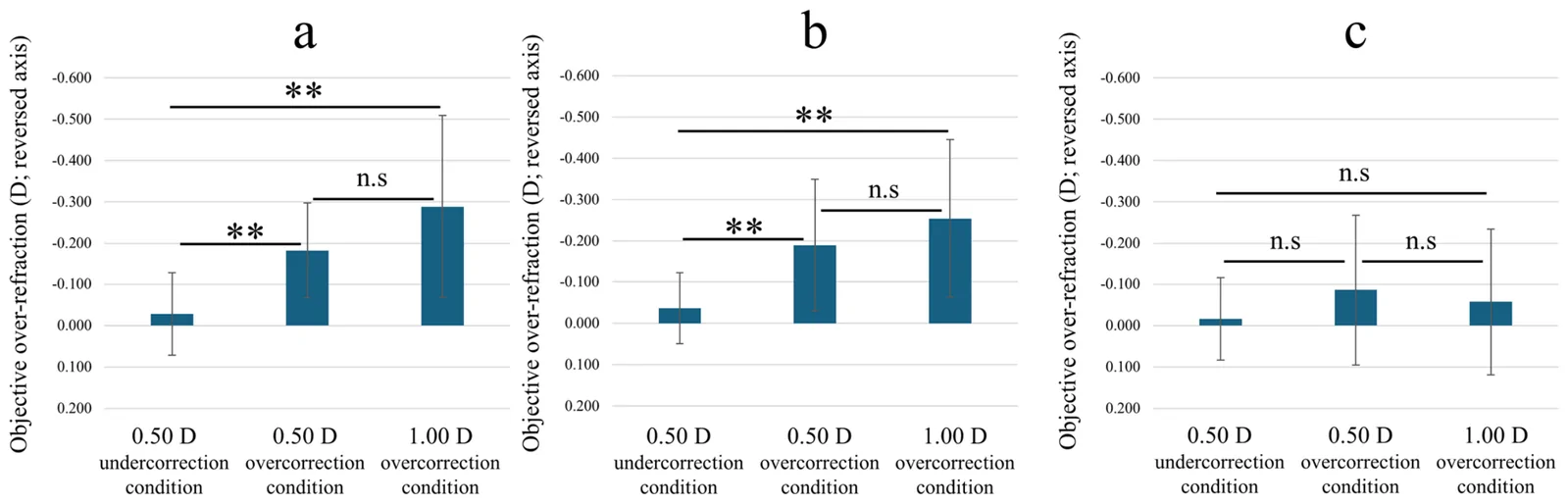
Objective over-refraction after restoration of the objective neutral correction at (**a**) 5 s, (**b**) 10 s, and (**c**) 30 s. Bars indicate mean values, and error bars indicate standard deviations. Negative values indicate residual objective myopic bias; the y-axis is reversed so that more negative values are plotted upward. Significance markers indicate Holm-adjusted paired comparisons within each time point. ** *p* < 0.01; n.s., not significant.

For the primary endpoint at 5 s after restoration of the objective neutral correction, mean OR was −0.028 ± 0.100 D after 0.50 D undercorrection, −0.182 ± 0.116 D after 0.50 D overcorrection, and −0.288 ± 0.221 D after 1.00 D overcorrection. Compared with 0.50 D undercorrection, mean OR was more negative by 0.154 D after 0.50 D overcorrection and by 0.260 D after 1.00 D overcorrection. The corresponding median differences were −0.179 D and −0.222 D, and the 95% confidence intervals for the mean differences did not include zero. These inter-condition differences were statistically significant after Holm adjustment (*p* = 0.007 and *p* = 0.003, respectively). The mean difference between 1.00 D and 0.50 D overcorrection was −0.106 D, with a 95% confidence interval that included zero and was not statistically significant after adjustment (*p* = 0.067; Table 1 and Figure 2a).

In the exploratory analysis at 10 s, mean OR was −0.036 ± 0.086 D after 0.50 D undercorrection, −0.189 ± 0.160 D after 0.50 D overcorrection, and −0.254 ± 0.191 D after 1.00 D overcorrection. Compared with 0.50 D undercorrection, mean OR was more negative by 0.153 D after 0.50 D overcorrection and by 0.218 D after 1.00 D overcorrection. The corresponding median differences were −0.107 D and −0.189 D, and the 95% confidence intervals for the mean differences did not include zero. These differences remained statistically significant after Holm adjustment (*p* = 0.003 and *p* = 0.001, respectively). The mean difference between 1.00 D and 0.50 D overcorrection was −0.065 D, with a 95% confidence interval that included zero, and was not statistically significant after adjustment (*p* = 0.350; Table 1 and Figure 2b).

In the exploratory analysis at 30 s, mean OR was −0.016 ± 0.099 D after 0.50 D undercorrection, −0.086 ± 0.182 D after 0.50 D overcorrection, and −0.058 ± 0.176 D after 1.00 D overcorrection. The corresponding mean differences were small: −0.071 D for 0.50 D overcorrection versus 0.50 D undercorrection, −0.042 D for 1.00 D overcorrection versus 0.50 D undercorrection, and 0.029 D for 1.00 D versus 0.50 D overcorrection. All 95% confidence intervals included zero, and none of the inter-condition differences were statistically significant after Holm adjustment (all *p* = 0.910; Table 1 and Figure 2c). In the post hoc power analysis, the observed standard deviations of the paired 30 s differences were 0.258 D, 0.178 D, and 0.258 D for the three comparisons, respectively. With *n* = 20, the power to detect a clinically meaningful paired mean difference of 0.25 D was 98.4%, >99.9%, and 98.4%, respectively, at α = 0.05. Even when using a conservative α level of 0.0167 for the three 30 s comparisons, the corresponding power values were 94.6%, >99.9%, and 94.6%. These results indicate that the study had sufficient sensitivity to detect a clinically meaningful residual difference at 30 s, supporting the interpretation that the remaining inter-condition differences at 30 s were clinically negligible.

The randomized condition order was balanced across the six possible sequences, with each sequence including three or four participants. In the exploratory sequence-effect analysis, no statistically significant period/order effects were found at 5, 10, or 30 s after restoration of the objective neutral correction (*p* = 0.356, *p* = 0.909, and *p* = 0.945, respectively). Similarly, no statistically significant previous-condition effects were found at 5, 10, or 30 s (*p* = 0.995, *p* = 0.090, and *p* = 0.089, respectively). These exploratory analyses did not indicate that measurement order or the immediately preceding lens condition materially influenced the OR outcomes.

**Figure 3:**
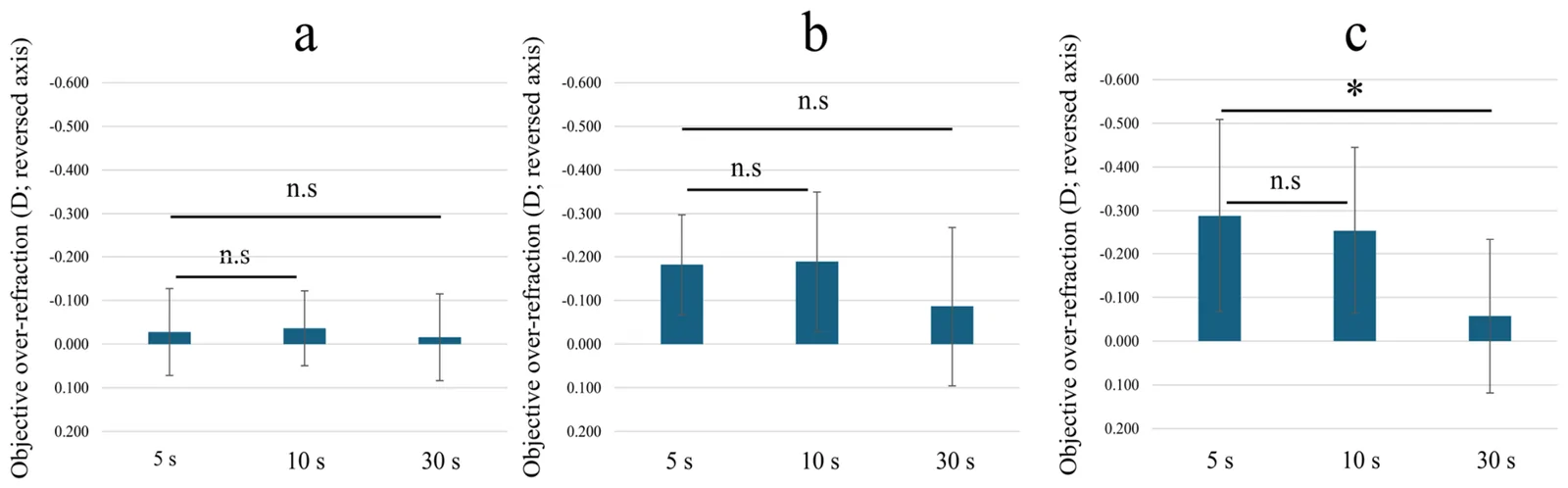
Within-condition changes in objective over-refraction from 5 to 10 s and from 5 to 30 s after restoration of the objective neutral correction. (**a**) 0.50 D undercorrection condition; (**b**) 0.50 D overcorrection condition; and (**c**) 1.00 D overcorrection condition. Bars indicate mean values, and error bars indicate standard deviations. Negative values indicate residual objective myopic bias; the y-axis is reversed so that more negative values are plotted upward. Significance markers indicate Holm-adjusted paired comparisons within each condition. * *p* < 0.05; n.s., not significant.

Within-condition OR did not differ significantly between 5 and 10 s or between 5 and 30 s after 0.50 D undercorrection (*p* = 0.65 and *p* = 0.59, respectively; Figure 3a) or after 0.50 D overcorrection (*p* = 0.88 and *p* = 0.06, respectively; Figure 3b). After 1.00 D overcorrection, OR became less negative from 5 to 30 s (*p* = 0.020; Figure 3c), whereas the change from 5 to 10 s was not statistically significant (*p* = 0.600).

## 4. Discussion

This study quantified the short-term residual objective myopic bias after a brief over-minus exposure during distance refraction. For the primary endpoint at 5 s after restoration of the objective neutral correction, 10 s of 0.50 D or 1.00 D myopic overcorrection produced more negative objective OR than did 0.50 D undercorrection. Exploratory analyses showed a similar pattern at 10 s, whereas the remaining inter-condition differences at 30 s were small, statistically non-significant, and clinically negligible rather than indicating complete physiological resolution. These results are consistent with a known slow component of accommodative relaxation rather than a new accommodative phenomenon. The main contribution of this study is therefore the clinical quantification of the magnitude and recovery time of this residual myopic bias after a brief overcorrection step. As the study measured objective refraction only, implications for subjective refraction strategy should be interpreted as inferential rather than as direct evidence of improved final subjective refractive outcomes. In clinical practice, these findings suggest that objective OR measurements obtained immediately after over-minus lens exposure should not be used directly to guide subsequent subjective refinement in young adults with myopia. To avoid or reduce this residual myopic bias, clinicians should minimize unnecessary over-minus exposure, return to the objective neutral correction or a slightly less-minus state, and allow a short period of distance fixation before relying on OR for further lens selection. Based on the present time course, waiting approximately 30 s after restoration of the objective neutral correction may be sufficient to reduce the bias to a clinically negligible level. Subsequent subjective refinement should then proceed according to the standard least-minus principle, using the least minus lens that provides the best visual acuity.

Accommodative relaxation is generally rapid in young adults, but may include a smaller, slower component that persists for several to tens of seconds [14,15]. This finding is consistent with the two-component (fast and slow) response [20,21]. The small residual myopic shift observed under overcorrection in this study likely reflects this slow component and is on the order of a single lens step, consistent with previous physiological reports [15]. Near-work-induced transient myopia (NITM) is a related phenomenon, with a mean residual myopia of approximately 0.4 D [22] that decays over several tens of seconds [23,24,25]. NITM typically follows sustained viewing of real near targets and subsequent distance fixation. Blur/distance cues and divergence-driven disaccommodation (via the vergence–accommodation cross-link) facilitate relaxation [26]. In the present study, the fellow eye (left) was occluded throughout each experimental condition, including both the 10 s lens-exposure period and the subsequent OR measurements, to approximate the monocular conditions commonly used during clinical distance refraction. Thus, accommodative relaxation was assessed under monocular viewing while the viewing distance remained fixed at 5 m. Under these conditions, binocular vergence cues were unavailable, and the absence of vergence-driven disaccommodation cues could theoretically have influenced the absolute magnitude or recovery time of the residual myopic bias. However, the occlusion procedure was identical across all three lens conditions, and the present data do not indicate that occlusion differentially altered the inter-condition comparisons. In addition, responses to lens-induced optical defocus may not be identical to accommodative responses elicited by real-space changes in viewing distance [27].

This study has some limitations, including the small sample size and sex imbalance. The cohort included only 20 participants (16 women and four men), and this limited sample size precluded sex-stratified analysis. Sex- or gender-related biological and physiological factors could theoretically influence accommodative behavior, including hormonal status, anterior-segment or iris morphology, and ciliary muscle characteristics. However, the present study did not assess menstrual cycle or hormonal status, iris morphology, ciliary muscle biometry, or other sex-related ocular biometric variables. Therefore, whether these factors contributed to inter-individual variability in the magnitude or recovery time of residual myopic bias could not be determined. The generalizability of the present findings, particularly to male participants, should therefore be interpreted with caution. Future confirmatory studies should include a larger and more sex-balanced cohort and consider measuring sex-related biological variables and ocular biometric parameters relevant to accommodation. Axial length was also not measured because the present pilot study focused on the short-term within-subject time course of objective refractive bias after brief over-minus exposure rather than on structural determinants of myopia. However, axial length may be associated with ciliary muscle morphology or accommodative dynamics, and the lack of axial length data limited our ability to examine whether ocular biometry contributed to inter-individual variability in residual myopic bias. Future studies should prioritize axial length measurement, ideally together with ciliary muscle or anterior-segment biometric assessment, to strengthen mechanistic interpretation. In addition, baseline accommodative and binocular vision metrics, including amplitude of accommodation, phoria status, and convergence measures, were not assessed. These variables could help explain inter-individual differences in the speed or completeness of accommodative relaxation after overcorrection. The absence of these measures therefore limited the mechanistic interpretation of individual variability in the present study. Future studies should include baseline accommodative and binocular function measures to clarify why some individuals show slower recovery after over-minus exposure. Additional limitations include the evaluation of only two levels of overcorrection (0.50 and 1.00 D), despite the possibility of larger residuals with stronger accommodative loads [28], and the observation period, which was limited to 30 s. Longer-term decay and cumulative effects with repeated testing were not assessed. Although all three conditions were completed in a single session, the order of the conditions was randomized, a 5 min rest period was provided between conditions, and OR was confirmed to have returned to approximately 0.00 D before the next condition was started. These procedures were intended to reduce carryover of accommodative state between conditions. In addition, the exploratory sequence-effect analysis showed no statistically significant period/order effects or previous-condition effects at 5, 10, or 30 s. These findings suggest that the randomized condition order and rest procedure minimized measurable carryover effects and that the main findings were not materially influenced by measurement sequence. Nevertheless, because this pilot study was not powered specifically to detect small sequence effects, subtle carryover effects cannot be definitively excluded. Finally, the present study assessed objective OR only and did not test whether the 5 s residual myopic bias caused measurable subjective visual acuity loss or altered final subjective refractive outcomes. Future studies should pair objective autorefraction with subjective visual acuity testing and subjective refraction endpoints to determine the functional significance of the short-term objective bias. The cohort also included only young adults with myopia, and further studies involving children, older adults, and individuals with different refractive error profiles are warranted to validate the generalizability of the findings.

## 5. Conclusions

This study quantified the short-term residual objective myopic bias after 10 s of transient 0.50 D or 1.00 D myopic overcorrection in young adults with myopia. The bias was evident at 5 and 10 s after restoration of the objective neutral correction, whereas the remaining inter-condition differences at 30 s were small, statistically non-significant, and clinically negligible. These findings characterize the magnitude and recovery time of a clinically relevant objective-refraction bias after brief over-minus exposure, rather than establishing a new physiological mechanism or directly testing final subjective refractive outcomes.

## Figures and Tables

**Table 1:** Primary and exploratory effect estimates for inter-condition differences in objective over-refraction after restoration of the objective neutral correction.

Time Point	Comparison	Mean Difference, D	Median Difference, D	95% CI for Mean Difference, D	Rank-Biserial r	Holm-Adjusted *p* Value
5 s	0.50 D overcorrection minus 0.50 D undercorrection	−0.154	−0.179	−0.231 to −0.075	−0.743	0.007
5 s	1.00 D overcorrection minus 0.50 D undercorrection	−0.260	−0.222	−0.372 to −0.146	−0.838	0.003
5 s	1.00 D overcorrection minus 0.50 D overcorrection	−0.106	−0.186	−0.214 to 0.011	−0.467	0.067
10 s	0.50 D overcorrection minus 0.50 D undercorrection	−0.153	−0.107	−0.233 to −0.078	−0.819	0.003
10 s	1.00 D overcorrection minus 0.50 D undercorrection	−0.218	−0.189	−0.304 to −0.136	−0.900	0.001
10 s	1.00 D overcorrection minus 0.50 D overcorrection	−0.065	−0.015	−0.188 to 0.056	−0.238	0.350
30 s	0.50 D overcorrection minus 0.50 D undercorrection	−0.071	−0.021	−0.187 to 0.034	−0.229	0.910
30 s	1.00 D overcorrection minus 0.50 D undercorrection	−0.042	−0.052	−0.118 to 0.033	−0.262	0.910
30 s	1.00 D overcorrection minus 0.50 D overcorrection	0.029	0.046	−0.083 to 0.138	0.171	0.910

Abbreviations: CI, confidence interval; D, diopter; and r, rank-biserial correlation coefficient.

## Data Availability

The data presented in this study are available from the corresponding author upon reasonable request. The data are not publicly available because of privacy and ethical restrictions related to participant data.

## Abbreviations

The following abbreviations are used in this manuscript:

CI	confidence interval
NITM	near-work-induced transient myopia
OR	objective over-refraction
SD	standard deviation
